# Early versus late closure of protective ileostomy after rectal cancer surgery

**DOI:** 10.1007/s00384-026-05239-y

**Published:** 2026-09-10

**Authors:** Elias Khajeh, Andrea Sosková, Johanna Fellhofer-Hofer, Nastaran Sabetkish, Rajan Nikbakhsh, Mohammadamin Shahrbaf, Arianeb Mehrabi, Fee Klupp

**Affiliations:** Department of General, Visceral and Transplantation Surgery, Heidelberg University, Heidelberg, Germany

**Keywords:** Colorectal Cancer, Protective Ileostomy, Morbidity, Early Closure, Late Closure, Ileostomy Reversal

## Abstract

**Purpose:**

The optimal timing of protective ileostomy closure after rectal cancer surgery remains uncertain. This systematic review and meta-analysis compares early (EC) versus late closure (LC) and evaluates the associated risks and benefits.

**Methods:**

Medline, Web of Science, and CENTRAL were searched for RCTs published up to May 2025 that compared EC (≤ 30 days after creation) with LC (≥ 12 weeks after creation) following rectal cancer surgery.

**Results:**

Nine RCTs including 387 patients (EC: 195, LC: 192) were analyzed. We found no significant difference between the two groups regarding intraoperative blood loss, operation time, length of hospital stay, overall postoperative morbidity, major complications, anastomotic leakage, readmission, perioperative mortality, administration of adjuvant chemotherapy, occurrence of LARS, and quality of life. However, the EC group showed a tendency toward better functional outcomes in terms of shorter time to first passage of flatus (*p* < 0.001). Additionally, one RCTs reported a significant, yet clinically irrelevant delay in initiation of adjuvant chemotherapy in the EC group (*p* < 0.001). Two RCTs demonstrated a significant cost reduction in the EC group – lower costs of stoma bags over the treatment period (*p* < 0.001) and lower total cost at 12 months following the initial rectal resection (*p* < 0.001).

**Conclusion:**

EC can be considered a safe and feasible alternative to LC in carefully selected patients. However, caution is required in treating high-risk patients, and further research is necessary in order to identify potential risk factors that may predict unfavorable outcomes.

**Supplementary Information:**

The online version contains supplementary material available at https://doi.org/10.1007/s00384-026-05239-y.

## Introduction

Over the past two decades, the incidence of early-onset colorectal cancer has increased. Tumors in younger patients predominantly arise in the distal colon and rectum. Rectal cancer treatment requires more aggressive treatment approaches due to the advanced stage [1–3]. Treatment planning depends on the location and stage of the rectal cancer. In early-stage rectal cancer with negative lymph node findings, surgery is performed as a first step. Patients with an advanced tumor e.g. T3-T4, and/or positive lymph nodes, initially receive neoadjuvant radiochemotherapy followed by surgery [4–7]. Robotic and laparoscopic surgery are nowadays mostly used for treatment of rectal cancer. Low anterior resection with total mesorectal excision (TME) is gold standard of surgical treatment for rectal cancer [8–10]. Anastomotic insufficiency is a serious complication after colorectal surgery. The incidence rate of anastomotic leakage ranges from 2 to 19% after rectal surgery [11]. It can lead to prolonged hospital stay, economical burden, increased morbidity and mortality as well as higher cancer recurrence rate. The mortality rate after anastomotic leakage following rectal surgery may reach 7% [12]. To reduce the risk of symptomatic anastomotic insufficiency, patients with low anastomoses near the pelvic floor often receive a protective ileostomy [13–15]. This can reduce the morbidity, reoperation rates, and mortality [14, 16, 17]. Nevertheless, timing of elective closure of protective ileostomy is controversial. Although some studies have been performed, there is still no clear evidence for the optimal time for stoma reversal. There are studies that support earlier reversal of protective ileostomy, demonstrating that early closure is safe and effective, and correlates with fewer adverse outcomes. On the other hand, late reversal has been also recommended in other studies in order to decrease the risk of anastomotic leakage.

Due to the discrepancies, a systematic review and meta-analysis was conducted to evaluate the advantages and disadvantages of both approaches as well as the best timing for protective ileostomy closure in order to minimize patient morbidity.

## Materials and methods

This systematic review was conducted in accordance with the Preferred Reporting Items for Systematic Reviews and Meta-Analyses (PRISMA) 2020 guidelines, as well as the recommendations of the Study Centre of the German Society of Surgery [18, 19]. The study was registered in Prospero: registration number CRD420261420911.

### Literature search

A systematic literature search was carried out in Medline (via PubMed), Web of Science, and CENTRAL databases based on the recommendations of Goossen et al. [20], using the following search terms:

((((("early"[Title/Abstract] OR “short-term” [Title/Abstract]) AND ("delayed"[Title/Abstract] OR "late"[Title/Abstract] OR "standard"[Title/Abstract])) AND ("ileostom*"[Title/Abstract] OR "enterostom*"[Title/Abstract] OR "surgical stoma*"[Title/Abstract])) AND ("reversal*"[Title/Abstract] OR "closure*"[Title/Abstract] OR "loop defunctioning*"[Title/Abstract] OR "defunctioning stoma*"[Title/Abstract])) AND ("low anterior resection*"[Title/Abstract] OR "high anterior resection*"[Title/Abstract] OR "sigmoid colectom*"[Title/Abstract] OR "distal colorectal resection*"[Title/Abstract] OR "rectal cancer*"[Title/Abstract] OR "colon cancer*"[Title/Abstract] OR "colon carcinoma*"[Title/Abstract] OR "rectal carcinoma*"[Title/Abstract] OR "colorectal carcinoma*"[Title/Abstract] OR "colorectal cancer*"[Title/Abstract] OR "rectal surger*"[Title/Abstract] OR "colon surger*"[Title/Abstract] OR "colorectal surger*"[Title/Abstract])) AND ("intervention study"[Title/Abstract] OR "controlled trial"[Title/Abstract] OR "randomized trial"[Title/Abstract] OR "clinical trial"[Title/Abstract] OR "RCT"[Title/Abstract] OR "parallel trial"[Title/Abstract] OR "trial"[Title/Abstract]).

The search strategy did not apply any publication date restrictions. The last search was performed on May 19, 2025.

### Eligibility criteria

The eligibility criteria were defined based on the Population, Intervention, Comparator, Outcome, and Study design (PICOS) approach.

- Population: Adult patients undergoing elective ileostomy closure following rectal surgery for rectal carcinoma.
- Intervention: Early ileostomy closure (EC), defined as closure performed within 30 days after protective ileostomy creation due to rectal cancer surgery.
- Comparator: Late ileostomy closure (LC), defined as closure performed 12 or more weeks after protective ileostomy creation due to rectal cancer surgery.
- Outcome: Intraoperative outcomes (including estimated blood loss and operation time), postoperative outcomes (including length of hospital stay, morbidity, readmission, mortality), adjuvant chemotherapy, time to initiation of adjuvant chemotherapy, functional outcomes, quality of life, costs.
- Study design: Randomized controlled trials (RCTs).

The exclusion criteria were studies on non-human populations, non-randomized studies, case reports, case series, conference papers, systematic reviews and meta-analyses. Moreover only studies with primary rectal cancer patients were included, patients with chronic inflammatory bowel disease were excluded, only rectal cancer surgery with exclusion of other operations like proctocolectomy. This criteria were determined in order to create a homogenous study collective as well as to reduce the risk of bias.

### Study selection and data extraction

Two investigators (AS and MAS) independently conducted the initial literature search using the predefined search terms. Titles and abstracts of all retrieved records were screened for eligibility according to the criteria described above, and potentially relevant articles were selected for full-text review. Regarding non-English publications, full texts were translated using electronic tools if the title and/or abstract matched the predefined inclusion criteria. Full-text articles of all studies identified as relevant were screened in detail, and data were extracted independently by two reviewers (AS and JFH). Secondary analyses of included RCTs were also considered if they reported outcomes that differed from those in the primary publications. Any discrepancies during the selection process or data extraction were resolved through consultation with the first author or senior author.

### Definition of extracted data

- Study characteristics: First author, year of publication, country of origin, study design, study period, follow-up duration, type of rectal surgery, diagnostic methods used before protective ileostomy closure, definitions of early and late ileostomy closure, and number of participants in each group.
- Patients’ characteristics: Age, gender, neoadjuvant therapy, and time to protective ileostomy closure.
- Intraoperative outcomes: Estimated blood loss and operation time.
- Postoperative outcomes: Length of hospital stay, overall complications, major complications (defined as Clavien-Dindo grade ≥ 3a), anastomotic leakage, readmission, mortality, adjuvant chemotherapy, and time to initiation of adjuvant chemotherapy.
- Functional outcomes: Time to first passage of flatus, time to first defecation, time to resumption of full oral intake, and Low Anterior Resection Syndrome (LARS).
- Quality of life: Gastrointestinal Quality of Life Index (GQLI) and EORTC QLQ-C30.
- Costs: Protective ileostomy-related costs and total costs.

### Quality assessment and risk of bias

The risk of bias of the RCTs included in the quantitative analysis was independently assessed by two investigators (AS and JFH) using the Cochrane Risk of Bias Tool version 2 (RoB 2) [21]. The quality of evidence for each outcome was evaluated by the same authors using the Grading of Recommendations, Assessment, Development, and Evaluation (GRADE) approach [22]. The first and senior authors were involved in resolving any disagreements arising during the abovementioned assessments.

### Statistical analysis

Statistical analysis was conducted using Review Manager Software (version 5.4.1; The Nordic Cochrane Centre, Cochrane Collaboration, Copenhagen, Denmark). Odds ratios (ORs) with 95% confidence intervals (95% CIs) were calculated for binary variables using the Mantel–Haenszel method, and mean differences (MDs) with 95% CIs were calculated for continuous variables using the inverse variance weighting. Only variables reported in at least two studies were included in the meta-analysis. Statistical heterogeneity was estimated using the I^2^ index and the Chi-square test. An I^2^ value > 50% and/or a *p* value for the Chi-square test < 0.1 were considered as indicators of substantial heterogeneity. To account for heterogeneity among the included studies, a random-effects model was applied. In addition, a sensitivity analysis was performed including only studies that defined early ileostomy closure as closure within two weeks after the index operation. Statistical significance was defined as *p* value < 0.05 for all analyses.

## Results

### Literature search

**Fig. 1:**
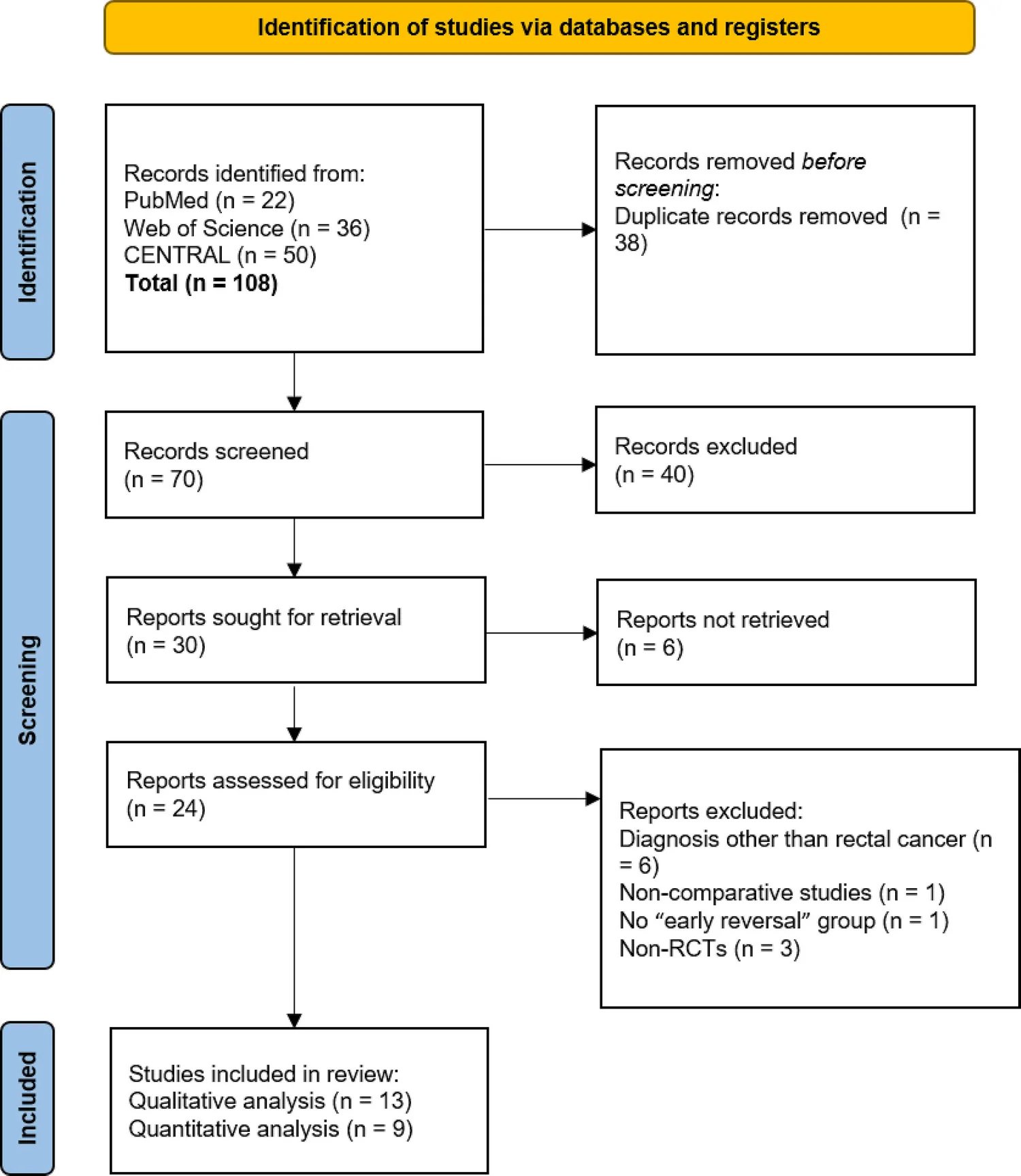
Search and selection process of the included studies. PRISMA flow chart

The systematic literature search initially identified a total of 108 articles. After removing duplicates, titles and abstracts of 70 studies were screened for eligibility, of which 24 articles were selected for further full-text review (Fig. 1).

Overall, thirteen studies enrolled exclusively patients with rectal cancer, and four studies were excluded from the quantitative analysis. In three of the four excluded studies, the definitions of early or late closure of the protective ileostomy were inconsistent with our definitions. In the study by Ahmadi-Amoli et al. [23], protective ileostomy reversal in the early closure group was performed 2–3 weeks after the first two courses of adjuvant chemotherapy, at an average of 9.14 weeks after protective ileostomy creation. In the study by Meyer et al. [24], early ileostomy closure was defined as closure performed less than three months after rectal surgery. In the study by Velagala et al. [25], protective ileostomy closure performed at six or more weeks after creation was considered late ileostomy reversal. Lastly, the study by Ellebaek et al. [26] was also excluded, as only 69% of patients assigned to the early ileostomy closure group actually received the intended intervention.

In total, nine studies were included in the quantitative analysis. Only studies with a definition of early ileostomy closure within 30 days after the operation were included. A detailed overview of the selection process is depicted in the PRISMA flowchart (Fig. 1).

### Methodological characteristics and quality of studies

The nine included studies [4, 8, 13, 27–32] were randomized controlled trials (RCTs) conducted in six countries and published between 2017 and 2021. The methodological characteristics of the included studies are summarized in Table 1.

**Table 1:** Methodological characteristics of the included studies

Study (Year)	Country	Period of the study	Pre-reversal diagnostics	Definition of EC	Definition of LC	Follow-up duration (months)
Danielsen et al. (2017) [27]	Denmark Sweden	2011–2015	contrast CT-scan and/or endoscopy	8–13 days	> 12 weeks	12
Park et al. (2018) [8]						12
Keane et al. (2019) [30]						52 (EC) 49 (LC)
Park et al. (2020) [13]						12
Klek et al. (2018) [4]	Poland	2016–2017	saline-flushing of the distal loop and endoscopy	18–20 days	7 months	NA
Bausys et al. (2019) [28]	Lithuania	2011–2017	retrograde contrast proctography and/or endoscopy	30 days	90 days	1
Dulskas et al. (2021) [31]						36
Gallyamov et al. (2019) [29]	Russia	NA	CT-proctography or rectoscopy	8–13 days	> 12 weeks	NA
Elsner et al. (2021) [32]	Switzerland	2007–2014	palpation, contrast enema, proctoscopy	2 weeks	12 weeks	4

*EC* Early closure, *LC* Late closure

**Fig. 2:**
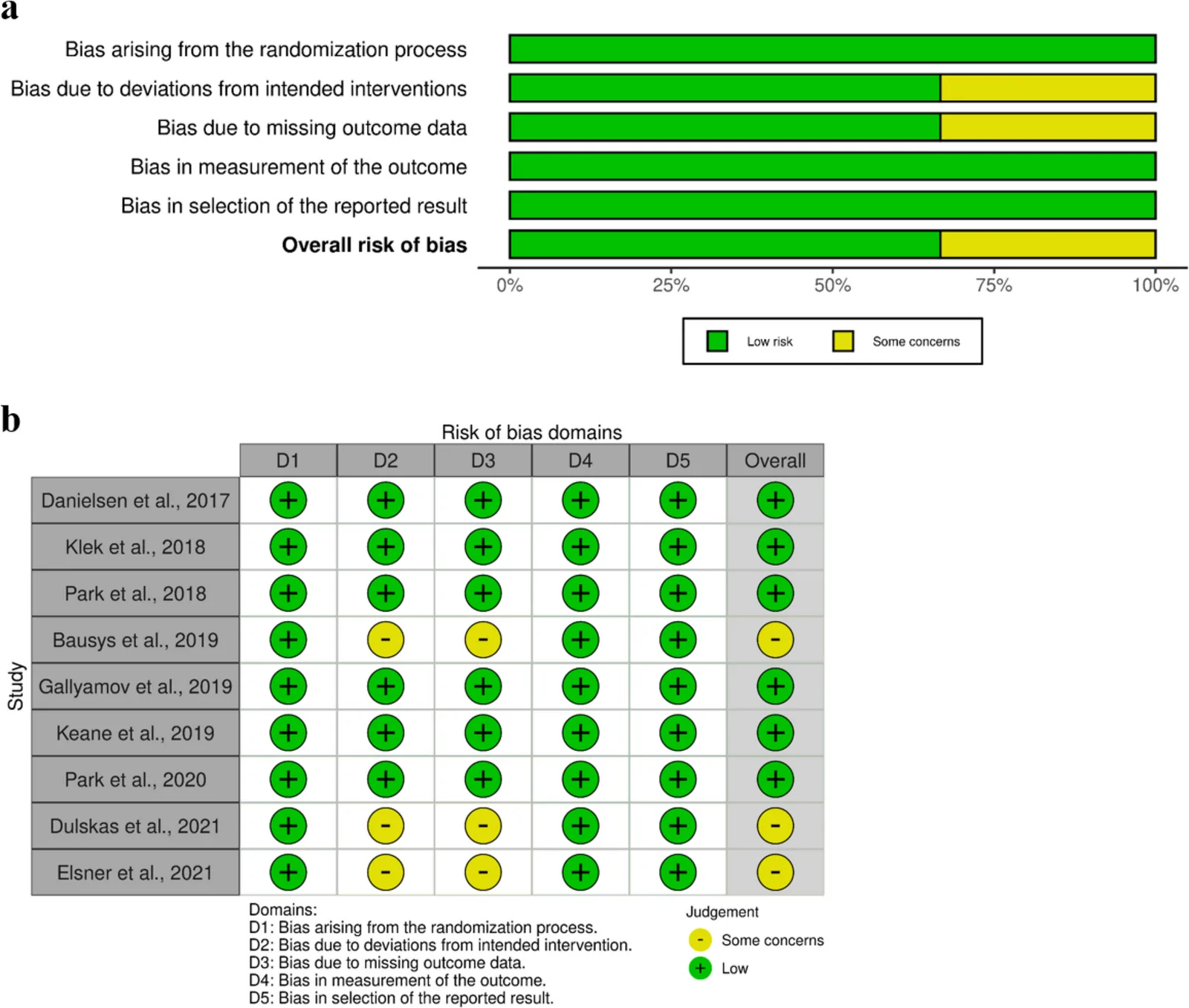
Risk of bias analysis of the included studies. **a**: risk of bias summary, **b**: risk of bias graph

Six of the included studies were assessed to have a low risk of bias, while three studies raised some concerns regarding methodological quality. None of the included studies were judged to have a serious risk of bias. In the “deviations from intended interventions” as well as the “missing outcome data” domain, six studies were evaluated to have a low risk of bias and three studies to raise some concerns due to the premature termination of the reported trials. All of the included studies were judged to have a low risk of bias in the domains “randomization process”, “measurement of the outcome” and “selection of the reported result”. A detailed assessment of the risk of bias for each study is provided in Fig. 2.

### Patients’ characteristics

In total, 387 patients were included in the meta-analysis, of which 195 patients underwent early ileostomy reversal and 192 patients underwent late ileostomy reversal. Table 2 provides an overview of the baseline characteristics of participants from each study.

**Table 2:** Patients’ characteristics

Study (Year)	Number of participants			Age (mean ± SD)		Gender (M/F)		Neoadjuvant therapy		Time to ileostomy reversal (days; mean ± SD)	
	EC	LC	Total	EC	LC	EC	LC	EC	LC	EC	LC
Danielsen et al. (2017) [27]	55	57	112	67 ± 11.5	67 ± 10.5	24/31	36/21	16	16	11 ± 36	148 ± 150.25
Park et al. (2018) [8]	55	57	112								
Keane et al. (2019) [30]	42	40	82	67 ± 13.3	68 ± 7.4	20/22	28/12			11 ± 2.96	150 ± 111.85
Park et al. (2020) [13]	55	57	112								
Klek et al. (2018) [4]	29	29	58	55.7 ± 12.2	56.2 ± 12.5	18/11	16/13	29	29	17.3 ± 1.5	278.6 ± 89.1
Bausys et al. (2019) [28]	43	38	81	65 ± 11.9	66 ± 7.4	25/18	18/25	20	19	34 ± 13.33	92 ± 57.04
Dulskas et al. (2021) [31]	26	25	51	63 ± 9.4	65 ± 9.3	14/12	11/14	14	13	34 ± 15	92 ± 25
Gallyamov et al. (2019) [29]	31	34	65	62 ± 11.7	67 ± 10.7	14/17	21/13	9	9	11 ± 3.32	148 ± 51.28
Elsner et al. (2021) [32]	37	34	71	67 ± 11.8	67 ± 9.8	21/16	26/8	13	12	15 ± 31	89 ± 13

*EC* Early closure, *LC* Late closure

### Quantitative analysis

#### Intraoperative outcomes

##### Intraoperative blood loss

**Fig. 3:**
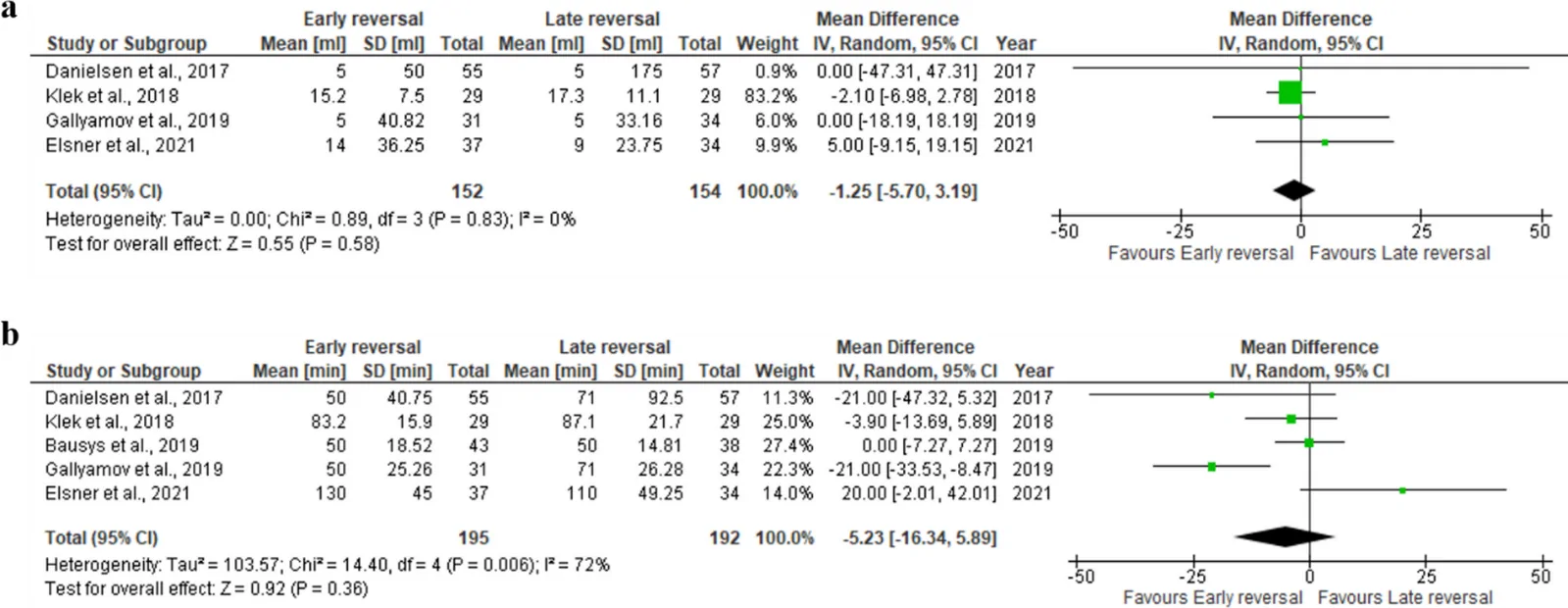
Intraoperative outcomes. **a**: intraoperative blood loss, **b**: operation time

Estimated intraoperative blood loss was reported in four studies comprising a total of 306 patients. The meta-analysis indicated a marginally lower blood loss in the early closure (EC) group, though the difference was not statistically significant (MD = −1.25 mL; 95% CI: −5.70 to 3.19; *p* = 0.580; GRADE: low). No heterogeneity was observed among these studies (I^2^ = 0%; *p* = 0.830) (Fig. 3a).

##### Operation time

Five studies including a total of 387 participants provided data on operation time. The meta-analysis showed no difference in operation time between the two groups (MD = −5.23 min; 95% CI: −16.34 to 5.89; *p* = 0.360; GRADE: low). Substantial heterogeneity was found among these studies (I^2^ = 72%, *p* = 0.006) (Fig. 3b). After excluding the study by Gallyamov et al. [29], the sensitivity analysis also showed no statistically significant difference between the groups (MD = −0.77 min; 95% CI: −10.32 to 8.78; *p* = 0.870). Substantial heterogeneity was still present among the studies (I^2^ = 51%, *p* = 0.110).

#### Postoperative short-term outcomes

##### Length of hospital stay

**Fig. 4:**
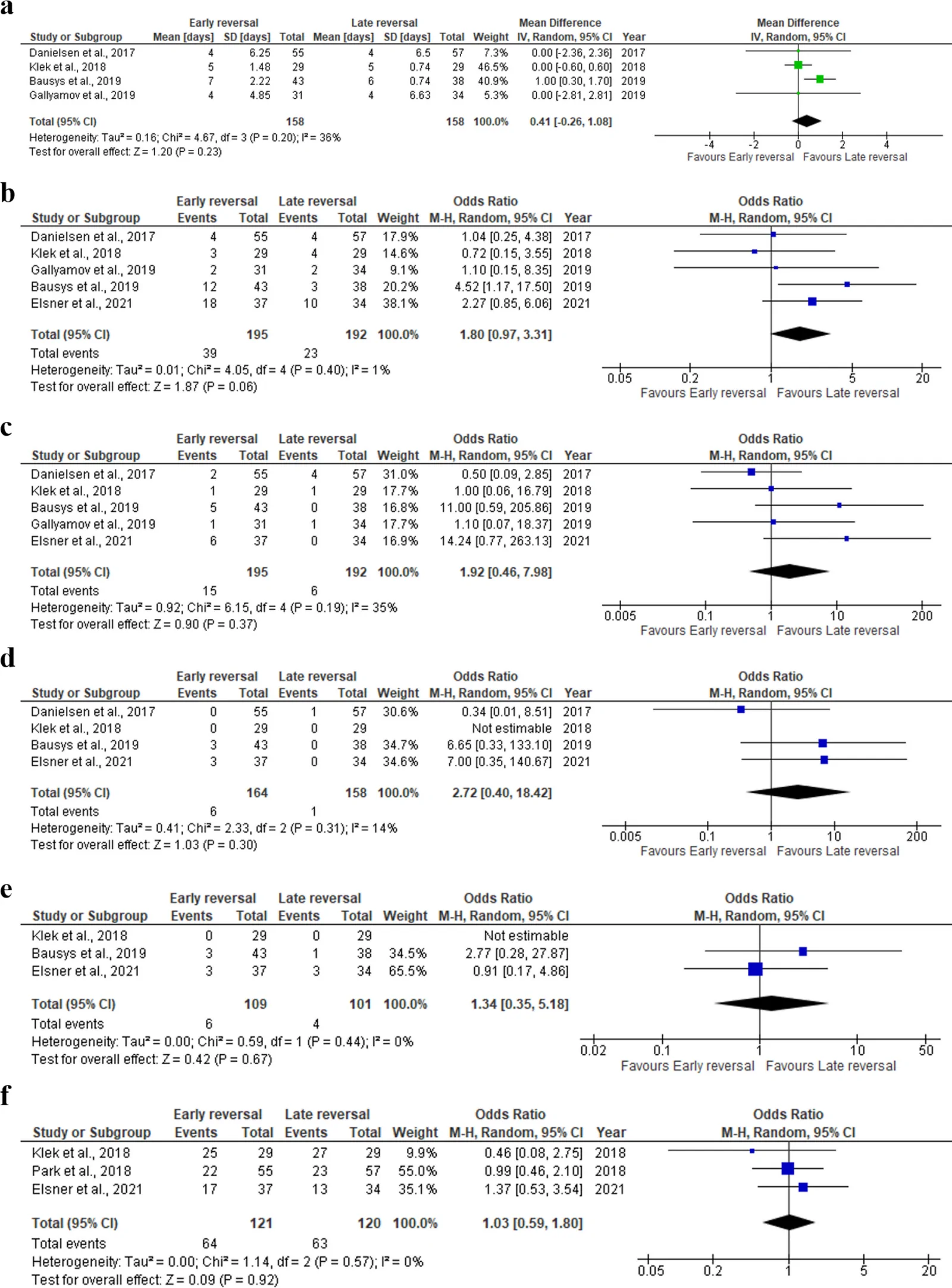
Postoperative outcomes. **a**: length of hospital stay following ileostomy closure, **b**: overall complications, **c**: major complications, **d**: anastomotic leakage, **e**: readmission, **f**: adjuvant chemotherapy

Four studies comprising 316 patients assessed length of hospital stay after protective ileostomy closure. The pooled analysis revealed a slightly longer postoperative stay in the EC group compared to the late closure (LC) group. However, no statistically significant difference was observed between the two groups (MD = 0.41 days; 95% CI: −0.26 to 1.08; *p* = 0.230; GRADE: low). There was a low level of heterogeneity among these studies (I^2^ = 36%; *p* = 0.200) (Fig. 4a).

##### Overall complications

Five studies including all 387 patients presented findings on overall morbidity. The overall complication rate was 20.0% (39/195) in the EC group compared to 12.0% (23/192) in the LC group. The results of the conducted meta-analysis did not indicate a statistically significant difference between the groups (OR = 1.80; 95% CI: 0.97 to 3.31; *p* = 0.060; GRADE: moderate). Very low heterogeneity was observed among the studies (I^2^ = 1%; *p* = 0.400) (Fig. 4b).

##### Major complications

Major complications, defined as Clavien-Dindo grade ≥ 3a, were assessed in five studies involving a total of 387 participants. The major complication rates were 7.7% (15/195) in the EC group and 3.1% (6/192) in the LC group. The pooled analysis showed no statistically significant difference between the two groups (OR = 1.92; 95% CI: 0.46 to 7.98; *p* = 0.370; GRADE: low). Low heterogeneity was detected among these studies (I^2^ = 35%; *p* = 0.190) (Fig. 4c).

##### Anastomotic leakage

Four studies comprising 322 patients reported on rates of leakage of the primary colorectal anastomosis following ileostomy closure. In the EC group, anastomotic leakage occurred in 3.7% (6/164) of cases compared to 0.6% (1/158) of cases in the LC group. The difference between the groups was not found to be statistically significant (OR = 2.72; 95% CI: 0.40 to 18.42; *p* = 0.300; GRADE: low). Low heterogeneity was observed across the reporting studies (I^2^ = 14%; *p* = 0.310) (Fig. 4d).

##### Perioperative mortality

Bausys et al. [28] provided data on postoperative 30-day mortality after protective ileostomy closure. No postoperative deaths were reported in the EC and the LC group. No postoperative deaths were reported in the EC and the LC group. The study by Ellebæk et al. [26] also reported no postoperative deaths within 30 days after protective ileostomy closure in both groups.

#### Long-term outcomes

##### Readmission

Readmission after protective ileostomy closure was assessed in three studies with a total of 210 participants. The readmission rate was 5.5% (6/109) in the EC group and 4.0% (4/101) in the LC group. The meta-analysis did not indicate a statistically significant different between the groups (OR = 1.34; 95% CI: 0.35 to 5.18; *p* = 0.670; GRADE: low). There was no heterogeneity among the included studies (I^2^ = 0%; *p* = 0.440) (Fig. 4e).

##### Adjuvant chemotherapy

Three studies comprising 241 participants provided data on adjuvant chemotherapy. In the EC group, adjuvant chemotherapy was administered to 52.9% (64/121) of patients, and in the LC group to 52.5% (63/120) of patients (OR = 1.03; 95% CI: 0.59 to 1.80; *p* = 0.920; GRADE: moderate). No evidence of heterogeneity was found among the included studies (I^2^ = 0%, *p* = 0.570) (Fig. 4f).

The study by Klek et al. [4] evaluated time to start of adjuvant chemotherapy, which was longer in the EC group, with a mean of 38.7 ± 5.7 days, compared to 33.2 ± 5.8 days in the LC group (*p* < 0.001).

#### Functional outcomes

**Fig. 5:**
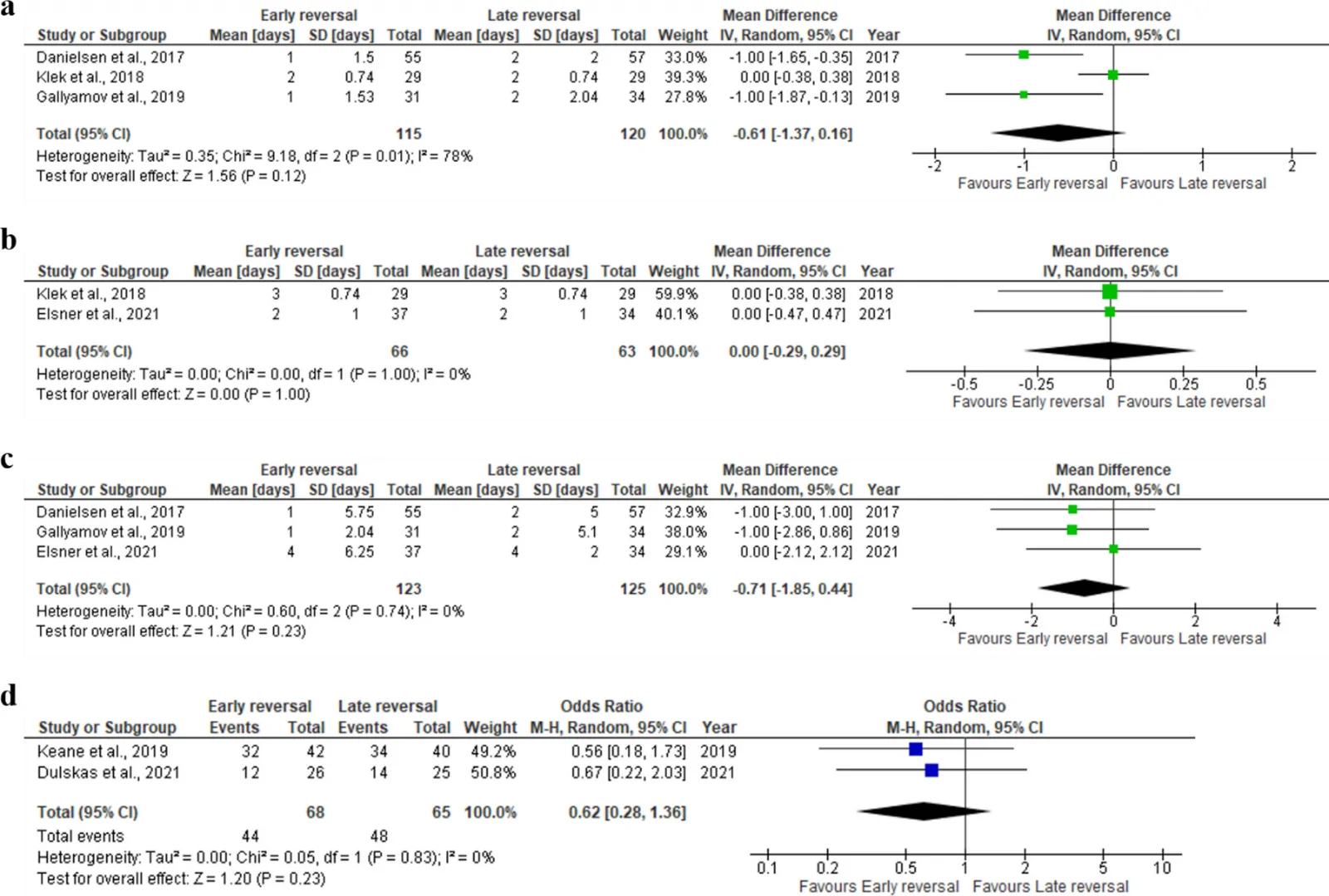
Functional outcomes. **a**: time to first passage of flatus, **b**: time to first defecation, **c**: time to resumption of full oral intake, **d**: Low Anterior Resection Syndrome

Three studies including 235 participants evaluated the time to first passage of flatus after protective ileostomy reversal. There was no difference between the groups (MD = −0.61 days; 95% CI: −1.37 to 0.16; *p* = 0.120; GRADE: very low). Substantial heterogeneity was found among these studies (I^2^ = 78%; *p* = 0.010) (Fig. 5a). However, the sensitivity analysis excluding the study by Klek et al. [4] showed a shorter time until first passage of flatus in the EC group (MD = −1.00 days; 95% CI: −1.52 to −0.48; *p* < 0.001) and there was no heterogeneity between the studies (I^2^ = 0%; *p* = 1.000).

Two studies with a total of 129 patients reported the time to first defecation following protective ileostomy closure. The analysis of pooled data did not reveal any difference between the EC group and the LC group (MD = 0.00 days; 95% CI: −0.29 to 0.29; *p* = 1.000; GRADE: low). No heterogeneity was detected among these studies (I^2^ = 0%; *p* = 1.000) (Fig. 5b).

The time to resumption of full oral intake after ileostomy reversal was assessed in three studies comprising 248 participants. The meta-analysis demonstrated no difference regarding return to full diet between the groups (MD = −0.71 days; 95% CI: −1.85 to 0.44; *p* = 0.230; GRADE: low). No evidence of heterogeneity was observed among the studies (I^2^ = 0%; *p* = 0.740) (Fig. 5c).

Two studies including a total of 133 patients provided data on Low Anterior Resection Syndrome (LARS) following ileostomy closure. LARS was observed in 64.7% (44/68) of patients in the EC group and in 73.8% (48/65) of patients in the LC group (OR = 0.62; 95% CI: 0.28 to 1.36; *p* = 0.230; GRADE: low). There was no heterogeneity among the reporting studies (I^2^ = 0%; *p* = 0.830) (Fig. 5d).

#### Quality of life

Two studies assessed global quality of life (GQoL) using the EORTC QLQ-C30 questionnaire. In the study by Park et al. [8], GQoL at 3, 6, and 12 months after rectal resection was comparable in the EC and LC group. In the study by Dulskas et al. [31], GQoL was also evaluated at a median of 36 months after ileostomy closure, showing no significant difference between the two groups.

The GQLI questionnaire was used to evaluate quality of life (QoL) in two studies. The results of the study by Elsner et al. [32] demonstrated comparable QoL in the EC and LC group at both 6 weeks as well as 4 months after rectal resection. The study of Ellebæk et al. [26] also reported no significant difference in mean QoL between the two groups at 6 months and at 12 months after rectal surgery.

#### Costs

Park et al. [13] reported a significantly lower total cost at 12 months following the initial rectal resection in the EC group, with a reduction in mean cost per patient of USD 4060 (95% CI: 1121 to 6999; *p* < 0.001). The study by Klek et al. [4] analyzed mean health care costs, defined as the costs of stoma bags over the treatment period, which were significantly lower in the EC group (PLN 152.9 ± 16.3; USD 43.68) compared to the LC group (PLN 2413.1 ± 759; USD 698.42; *p* < 0.001).

#### Sensitivity analysis of studies defining EC as closure within two weeks

A sensitivity analysis including only studies that defined early ileostomy closure as closure within two weeks after the index operation (n = 6) showed no statistically significant differences between the EC and LC groups regarding intraoperative blood loss (MD = 2.95 ml; 95% CI: −7.92 to 13.82; *p* = 0.590; I^2^ = 0%; *p* = 0.910), operative time (MD = −7.74 min; 95% CI: −34.11 to 18.64; *p* = 0.570; I^2^ = 81%; *p* = 0.005), length of hospital stay (MD = 0.00 days; 95% CI: −1.81 to 1.81; *p* = 1.000; I^2^ = 0%; *p* = 1.000), postoperative morbidity (OR = 1.66; 95% CI: 0.78 to 3.52; *p* = 0.190; I^2^ = 0%; *p* = 0.620), major postoperative complications (OR = 1.58; 95% CI: 0.21 to 11.89; *p* = 0.660; I^2^ = 51%; *p* = 0.130), anastomotic leakage (OR = 1.63; 95% CI: 0.08 to 32.05; *p* = 0.750; I^2^ = 45%; *p* = 0.180), and administration of adjuvant chemotherapy (OR = 1.12; 95% CI: 0.62 to 2.02; *p* = 0.700; I^2^ = 0%; *p* = 0.590) (Supplementary Fig. 1 and 2). However, patients in the EC group experienced a significantly shorter time to first passage of flatus compared to patients in the LC group (MD = −1.00 days; 95% CI: −1.52 to −0.48; *p* < 0.001; I^2^ = 0%; *p* = 1.000) (Supplementary Fig. 3).

## Discussion

The optimal timing of protective ileostomy closure after rectal cancer surgery remains a subject of ongoing debate – with controversial evidence as to whether early closure can be considered as a safe and feasible alternative to late closure. While some RCTs reported significantly shorter operation time and lower healthcare costs in the EC group, as well as comparable postoperative morbidity between the EC and LC groups [4, 13, 26, 27, 29], other RCTs were prematurely terminated due to dramatically higher complication and anastomotic leakage rates in the EC group [28, 32].

In the context of these conflicting findings, we conducted the present meta-analysis to compare the intraoperative, postoperative, functional and cost outcomes between early and late ileostomy closure in patients undergoing rectal cancer surgery. The objective was to evaluate the advantages and disadvantages of each approach in order to identify the optimal timing for ileostomy closure. This systematic review and meta-analysis revealed a tendency toward better functional outcomes in the EC group. However, longer time to initiation of adjuvant chemotherapy in the EC group has been reported [4], while EC can reduce the postoperative costs significantly [4, 13]. Overall, no statistically significant differences were observed between the EC and LC groups in terms of intraoperative blood loss, operation time, length of hospital stay, overall postoperative morbidity, major complications, anastomotic leakage, readmission, perioperative mortality, administration of adjuvant chemotherapy, occurrence of LARS, or quality of life.

We observed no significant difference in intraoperative blood loss between the two groups. We also found no significant difference in operation time between the two groups. Our findings are consistent with a previous systematic review that also demonstrated no difference in operative time between EC and LC group [33]. In contrast, another meta-analysis, that included patients with benign as well as malign disease, demonstrated a significantly shorter operation time in the EC group [34].

Our results did not show any difference between the two groups regarding length of hospital stay after ileostomy closure. Similar findings were presented in previous systematic reviews [33, 34]. However, the RCT by Bausys et al. [28] reported a significantly longer hospital stay in the EC group which may be explained by the higher postoperative morbidity observed in that cohort.

We observed no significant difference in postoperative morbidity, major complication rates and anastomotic leakage rates between the two groups. Although not statistically significant, our results showed tendency towards slightly higher overall complication rate in EC group, but no difference in major complications. Comparable morbidity and occurrence of anastomotic leakage in EC and LC group have already been reported in previous meta-analyses [33–36]. These studies also suggest that EC may be associated with more wound complications or infections, whereas small bowel obstruction and stoma-related complications appear less frequent in this cohort. Regmi et al. [37] stated no higher morbidity rates after early closure of ileostomy, but it has to be mentioned that studies with patients with underlying benign diseases were also included in their study and additionally the definition of EC was up to 3 months in this meta-analysis. However, a recently published meta-analysis by Emile et al. [38] reported significantly higher anastomotic leakage rates following early ileostomy closure, particularly when closure was performed within two weeks after the index operation. These discrepant findings may be explained by methodological differences to our meta-analysis, such as the inclusion of patients with benign disease, heterogeneous definitions of early closure, and the incorporation of trials with limited adherence to the allocated intervention. In contrast, our meta-analysis applied a strict definition of early closure, included only randomized controlled trials enrolling patients with rectal cancer, and excluded studies with substantial protocol deviations. Nevertheless, it has to be discussed, that there are no current guidelines which mandatorily prescribe the necessarily needed diagnostic tools prior to protective ileostomy closure. Therefore, different strategies like contrast enema, proctography, endoscopy, rectoscopy, palpation or saline testing exist in order to ensure the integrity of the anastomosis before ileostomy closure. Regardless of the method used, a reliable assessment of the anastomosis is crucial prior the protective ileostomy closure. However, differences in pre-closure assessments may partly explain heterogeneity in outcomes. When considering these methodological aspects, our findings support the conclusions of earlier meta-analyses and suggest that early ileostomy closure, when performed in carefully selected patients and at an appropriate time point, does not increase the risk of anastomotic leakage or major postoperative complications.

Notably, the EASY trial by Danielsen et al. [27] demonstrated a significantly lower mean number of complications at 12 months after the index rectal surgery in the EC group, supporting EC as a safe and feasible alternative to LC. In contrast, two trials were prematurely terminated due to significantly higher postoperative morbidity and major complication rates in the EC group, raising concerns that EC may carry more risks than benefits for certain patients [28, 32]. A possible explanation for the deviation from the findings of other RCTs is that Bausys et al. [28] defined EC as closure performed 30 days after rectal surgery, which differs considerably from the definitions used in most other trials and may affect the postoperative outcomes. A subgroup analysis of a previous systematic review that defined EC as closure within 15 to 30 days after the index surgery also reported a higher complication rate in the EC group [36]. Ileostomy closure at 30 days after creation may represent an unfavorable timepoint, as severe peristomal adhesions typically develop three to six weeks after surgery [39]. Furthermore, the divergent results of the RCT by Elsner et al. [32] may be attributable to an early randomization of participants, before sufficient control of anastomotic integrity, whereas other studies randomized their patients only after an uneventful postoperative course and exclusion of anastomotic leakage [27–29].

We found that both readmission rates as well as perioperative mortality were comparable between the EC and LC group. Our findings are consistent with a previous systematic review that also reported no significant difference in readmission between the two groups [36]. Although this suggests that EC does not increase the risk of readmission or perioperative mortality, the results need to be interpreted with caution due to the small number of RCTs reporting these outcomes.

Our meta-analysis did not demonstrate a significant difference in the rates of adjuvant chemotherapy administration between the two groups. Interestingly, the RCT by Klek et al. [4] reported a statistically significant delay in the initiation of adjuvant chemotherapy in the EC group (38.7 vs. 33.2 days, *p* < 0.001). However, this difference may be considered clinically irrelevant, as adjuvant chemotherapy was initiated within 8 weeks after surgery in both groups, which is in accordance with recommendations from previous studies [40, 41].

The meta-analysis showed a tendency toward better functional outcomes in the EC group in terms of shorter time to first passage of flatus. These findings, however, need to be interpreted cautiously, as the sensitivity analysis included only two studies reporting this outcome. No significant differences between the groups were observed regarding the occurrence of LARS, and similar findings were also presented in the meta-analysis by Podda et al. [36]. Notably, another meta-analysis by Keane et al. [42] suggested that the presence of an ileostomy itself may increase the risk of developing LARS. Taking this into account, by reducing the overall time with an ileostomy performing an EC, this approach may offer a potential benefit for patients by lowering the risk of developing LARS. However, further evidence is necessary to confirm this association.

Because of the use of different questionnaires and scoring systems, as well as variability in the timing of quality-of-life assessments, a pooled analysis of the data could not be performed. Studies that assessed quality of life using the EORTC QLQ-C30 or the GQLI questionnaire found no statistically significance between the groups [8, 26, 31, 32]. Nevertheless, several studies have shown that protective ileostomy reversal generally improves patients’ quality of life, suggesting that EC may prolong the period during which patients experience a better quality of life [10, 43, 44].

Although a pooled analysis of healthcare costs was not feasible, two studies reported a significant cost reduction in the EC group [4, 13]. These results suggest that EC may be associated with lower healthcare expenditures. However, due to the small number of studies and variability in cost-reporting methods, further evidence is necessary to confirm the potential economic advantages of EC.

To further evaluate the impact of timing of early ileostomy closure, we performed a sensitivity analysis including only studies that defined early closure as closure within two weeks after the index operation. The results were consistent with those of the primary analysis, demonstrating no significant differences in overall and major postoperative morbidity, anastomotic leakage rates, or administration of adjuvant chemotherapy compared to late closure. In addition, patients undergoing EC showed better functional outcomes, suggesting faster recovery of bowel function than patients undergoing LC. Although the available evidence does not allow to determine a single optimal time point for ileostomy closure, these findings indicate that closure within two weeks may be a safe and feasible option in patients with adequately healed anastomosis.

This systematic review and meta-analysis has several strengths and provides information on various intraoperative and postoperative outcome data. For our meta-analysis, only RCTs were included to ensure a high level of evidence and minimize bias. Moreover, we included only RCTs reporting on patients with rectal cancer that underwent rectal surgery with a temporary ileostomy. Unlike previous systematic reviews, which also included studies with individuals diagnosed with benign disease, our meta-analysis focuses exclusively on patients diagnosed with rectal cancer.

Some limitations of our systematic review and metaanalysis has to be mentioned. Most importantly, the definitions of early ileostomy closure varied across studies, ranging from 8–12 days up to 30 days after the index rectal surgery, which contributed to heterogeneity among the included RCTs. An overall precise definition of early closure is necessarily needed because otherwise this can lead to different outcomes and results in higher study bias. Also, a clear definition of the time frame of late closure of protective ileostomy should be defined per official guideline. We stated late closure after at least 12 weeks after rectal cancer surgery with protective ileostomy and excluded earlier time points. Moreover, heterogenous preoperative investigations assessing the degree of the anastomotic recovery before protective ileostomy closure exist. Additionally, there was a lack of data for several outcomes – particularly functional outcomes, readmission, perioperative mortality, quality of life, and healthcare costs – limiting the possibility to perform pooled analyses for these endpoints. Therefore, further research is needed to better evaluate these outcomes.

## Conclusion

Our systematic review and meta-analysis showed no significant differences between EC and LC of protective ileostomy after rectal cancer surgery for most evaluated outcomes. However, we found a tendency toward better functional outcomes in the EC group. Significant delay in initiation of adjuvant chemotherapy in the EC group has been reported in one study, which can be considered as clinically not relevant, as adjuvant chemotherapy was nevertheless initiated within 8 weeks after surgery in both groups, as recommended by clinical guidelines. A significant cost reduction in the EC group was also found. In conclusion, these findings suggest that EC appears to be a feasible option in carefully selected patients, which may result in reduction of financial burden of health care systems. However, caution and careful consideration is required in treating high-risk patients, and further research is necessary in order to identify potential risk factors that may predict unfavorable outcomes and in order to evaluate the most suitable time point for protective ileostomy closure after rectal cancer surgery.

## Supplementary Information

Supplementary Material 1.

## Data Availability

Within original article.
