# Supplementary material for: Early versus late closure of protective ileostomy after rectal cancer surgery
Source: Int J Colorectal Dis. 2026 Sep 10;41(1):149. doi: 10.1007/s00384-026-05239-y (PMC13562142; doi:10.1007/s00384-026-05239-y)
Supplement: Supplementary file 1 [file 384_2026_5239_MOESM1_ESM.docx]

**Supplementary Fig. 1** Sensitivity analysis of EC ≤2 weeks – intraoperative outcomes. **a:** intraoperative blood loss, **b:** operation time

**Supplementary Fig. 2** Sensitivity analysis of EC ≤2 weeks - postoperative outcomes. **a:** length of hospital stay following ileostomy closure, **b:** overall complications, **c:** major complications, **d:** anastomotic leakage, **e:** adjuvant chemotherapy

**Supplementary Fig. 3** Sensitivity analysis of EC ≤2 weeks - functional outcomes. **a:** time to first passage of flatus, **b:** time to resumption of full oral intake
